# Assessing the Impact of Village Development on the Habitat Quality of Yunnan Snub-Nosed Monkeys Using the INVEST Model

**DOI:** 10.3390/biology11101487

**Published:** 2022-10-11

**Authors:** Shuxian Zhu, Li Li, Gongsheng Wu, Jialan Liu, Timothy J. Slate, Hongyan Guo, Dayong Li

**Affiliations:** 1 Key Laboratory of Southwest China Wildlife Resources Conservation (Ministry of Education), China West Normal University, Nanchong 637001, China; 2 Wildlife Management and Ecosystem Health Center, Yunnan University of Finance and Economics, Kunming 650221, China; 3 Natural Resources Bureau of Heping County, Heyuan 517200, China; 4 Kunming Institute of Zoology, Chinese Academy of Sciences, Kunming 650201, China

**Keywords:** snub-nosed monkey, village development, habitat quality, InVEST model, conservation policies, species conservation

## Abstract

**Simple Summary:**

The Yunnan snub-nosed monkey is one of the most endangered species on the IUCN Red List. The study of its population and habitat quality is important in identifying opportunities for balancing socio-economic development against species conservation in the area’s villages. Such balances are important to protecting and improving habitat diversity and biodiversity. Our habitat quality analysis indicates that increases in socio-economic developments in the villages around the habitat area have decreased both the habitat area and the habitat quality over time. This has resulted in a decline in biodiversity persistence, resilience, and breadth. It also has exacerbated the risk of declining species populations, potentially to extinction. Though focused on the Yunnan snub-nosed monkey, our approach toward the assessment of habitat quality based on species habitat suitability introduces a new perspective for assessing village development impacts on the habitat quality for the conservation of other species.

**Abstract:**

The habitats of the already endangered Yunnan snub-nosed monkey (*Rhinopithecus bieti*) are degrading as village economies develop in and around these habitat areas, increasing the depopulation and biodiversity risk of the monkey. The paper aims to show the areas of these monkeys’ high-quality habitats that are at highest risk of degradation by continued village development and hence be the focus of conservation efforts. Our analysis leveraged multiple tools, including primary component analysis, the InVEST Habitat-Quality model, and GIS spatial analysis. We enhanced our analysis by looking at habitat quality as it relates to the habitat suitability for the monkey specifically, instead of general habitat quality. We also focused on the impact of the smallest administrative scale in China—the village. These foci produced a clearer picture of the monkeys’ and villages’ situations, allowing for more targeted discussions on win–win solutions for both the monkeys and the village inhabitants. The results show that the northern habitat for the monkey is currently higher quality than the southern habitat, and correspondingly, the village development in the north is lower than in the south. Hence, we recommend conservation efforts be focused on the northern areas, though we also encourage the southern habitats to be protected from further degradation lest they degrade beyond the point of supporting any monkeys. We encourage developing a strategy that balances ecological protection and economic development in the northern region, a long-term plan for the southern region to reduce human disturbance, increase effective habitat restoration, and improve corridor design.

## 1. Introduction

Northwestern Yunnan, with its rich natural resources, is among the most biodiverse areas in China. It is home to one of the most endangered species on Earth—the Yunnan snub-nosed monkey [1,2]. Yunnan snub-nosed monkeys are scattered in the Three Parallel Rivers region in China, spending most of their time in the dense fir-dominated coniferous forests and mixed coniferous forests [1,2]. They remain on high alert for approaching humans, moving quickly to avoid them [3,4,5]. This makes conducting accurate surveys difficult. In 2016, 15 isolated groups totaling approximately 3000 individuals were surveyed by tracking and photographing the monkeys and recording GPS information of the tracking route [6].

The continuing development of villages around the monkey habitat, as well as other factors, have led to overlap between the villages and the monkey habitats [7,8]. Human activities in these overlapping villages, such as deforestation and land cultivation for agriculture to develop their economies, have impacted the monkey habitat. Increase in the monkey population is limited by the loss, degradation, and fragmentation of its habitat, and risk of decline in the monkey population is increased [3,4,5].

The plight of the endangered Yunnan snub-nosed monkey highlights the impact of the imbalance of socio-economic development versus ecological development in villages, with the socio-economic development currently winning out [3,5]. Studying this monkey’s habitat quality and population is an important foundation for balancing human economic development and species conservation in villages. This balance is an important way to protect an area’s habitat diversity and biodiversity [9,10].

Village development has increased human deforestation and use of land areas, including cultivating the land for agriculture and grazing, expanding urban boundaries, and introducing non-native invasive species [11,12,13]. Such activities led to the decline of habitat quality, the increase of habitat fragmentation, and even the outright loss of wildlife habitats [14], which, in turn, leads to reduced or lost regional biodiversity and the threat of wildlife depopulation [12,14,15,16]. Therefore, by promoting the coordinated development of socio-economic and habitat quality in villages, habitat quality will be improved, and the durability and stability of ecosystems will be maintained, thus protecting habitat diversity and biodiversity [17,18].

Being able to model habitat quality in an area effectively is critical as habitat quality—the ability of an ecosystem to provide for the survival, reproduction, and development of organisms—reflects biodiversity [19,20]. Habitat quality depends on the proximity of the habitat area to human-developed land and on the intensity of human land use, among other factors. Increases in intensity of human land use have been shown to degrade the quality of nearby natural habitats [12,21]. There has been significant research on the use of the InVEST (Integrated Valuation of Ecosystem Servicer and Trades) Habitat Quality model in assessing biodiversity and in developing conservation measures [22,23].

The InVEST-Habitat Quality model is based on habitat suitability, combined with land cover and biodiversity threat factors, to evaluate habitat quality [20,24]. For example, the InVEST model has been used for habitat quality studies assessing the spatial vulnerability of natural habitats in Chaharmahal and Bakhtriari provinces [25], of bird communities in Central Italy [26], of bird species in Keoladeo National Park [27], and of the wildlife habitat quality in the Greater Serengeti Ecosystem [28]. This model can also describe different threat factors of biodiversity, such as climate change, population density, road density, land use intensity, urbanization (village development and village agglomeration), and changes in landscape patterns [29,30]. This model allows comparison of spatial patterns of biodiversity and ecosystem services, prioritizing species populations for conservation by evaluating multiple land-use change scenarios to find scenarios that best take advantage of the conservation of nature reserves while benefiting human economic development [31].

Using the socio-economic data of 2572 villages and 344 sample plots in northwestern Yunnan, we used principal component analysis of GIS spatial analysis and the InVEST Habitat-Quality model to study the influence of village development on the habitat quality of Yunnan snub-nosed monkey distribution area in 2018, exploring these three challenges:

(1) Analyze and categorize the development status of villages;
(2) Analyze the spatial distribution of habitat quality in Yunnan snub-nosed monkey distribution area;
(3) With the results of those analyses, determine the impact of village development on the habitat quality of the Yunnan snub-nosed monkey population.

## 2. Materials and Methods

### 2.1. Study Area

**Figure 1:**
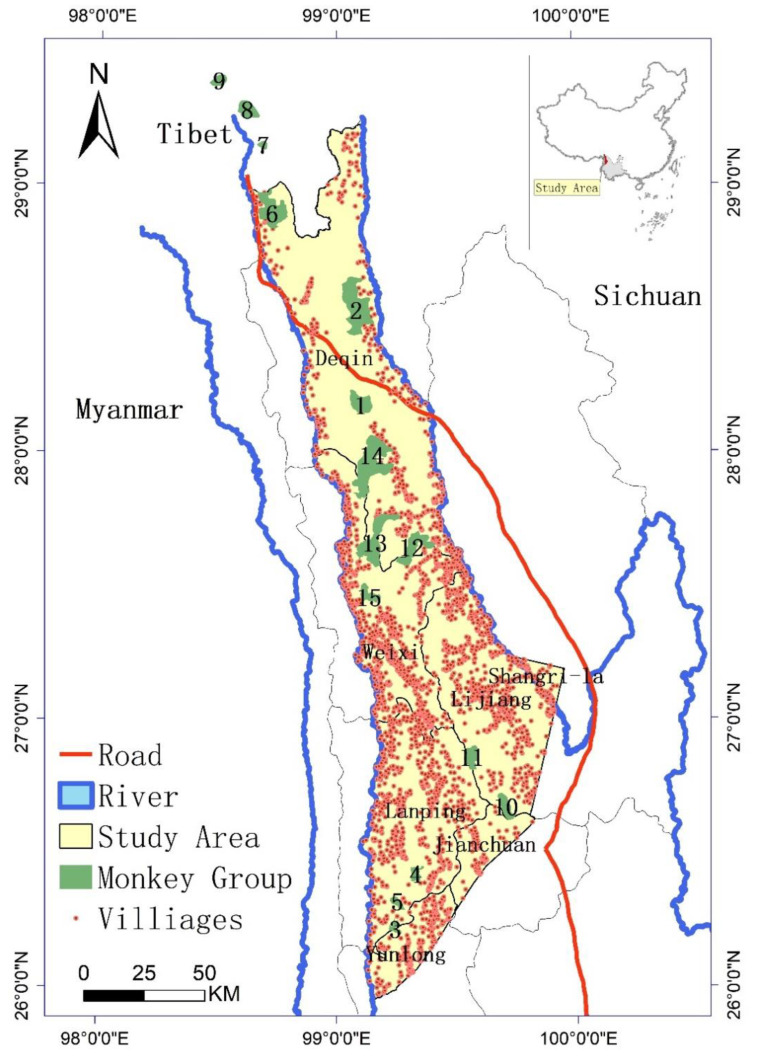
The study area and locations of monkey groups in Yunnan Province (China). The numbers labeling each green area represent the monkey group number (1–15).

The study area (Figure 1) is in the Three Parallel River region of northwest Yunnan Province (between 29.020 N, 98.030 E in the north and 25.053 N, 99.022 E in the south, with the elevation varying from 1200 m to 5500 m). It is one of the most ecologically significant areas of China in terms of biodiversity, covering approximately 17,000 km^2^ across seven counties in Yunnan (Deqin, Weixi, Lanping, Shangri-La, Lijiang, Jianchuan, and Yunlong), with a total human population of about 1,182,500.

The Yunnan snub-nosed monkey lives at a very high elevation (mainly above 3000 m) and is an endangered species on the IUCN Red List [32,33]; 15 groups of these monkeys have been identified previously. We obtained information on the monkey population size as of 2016, with the monkey population being approximately 3000 individuals at that time [6,32].

### 2.2. Land Use and Land Cover

Land Use and Land Cover (LULC) data in 2018 were obtained from a supervised classification on SPOT-5 images (Institute of Forest Inventory and Planning, Yunnan, 2012) with ground-truthing by the Conservation Information Centre of the Nature Conservancy’s China program. All data were geo-corrected in ERDAS 9.2 with a root-mean-square (RMS) error < 1. LULC types (such as Armand pine and hemlock) were assigned one of five habitat categories, based on the Yunnan vegetation classification system and the monkey’s habitat preferences. These five habitat categories, in declining quality, were optimal habitat, suboptimal habitat, suitable habitat, unfavorable habitat, and highly unfavorable habitat [5].

### 2.3. Villages and Rural Roads

Data on 2572 villages, related rural roads, and their socio-economic status were obtained from the National Geographic Information Resources Catalog Service system (https://www.webmap.cn/main/do?method=index, accessed on 13 December 2016) and Yunnan Digital Rural Network (http://www.ynszxc.gov.cn, accessed on 6 May 2018).

### 2.4. Principal Component Analysis

We selected 2572 villages located in the study area, obtaining 30 indicators for principal component analysis (Table 1). The 30 indicators were grouped into six categories: the natural resources, population, economy, infrastructure, energy, and educational factors. We used the principal component analysis [34] in R v.2.14.1 to calculate eigenvalues, using eigenvalues greater than 1 to identify the principal components of development factors of the village. This downscaled 30 variables into 8 principal components. We used weighted-sum method with these 8 principal components to calculate a comprehensive score for each village, which was used to evaluate the development level of village. Villages were then assigned into one of seven grades (I, II, III, IV, V, VI, and VII) using the Equal Interval Breaks Method (Table 2). Villages of grade I had the lowest village development, villages of grade VII had the highest.

### 2.5. Plots

The 344 plots were obtained from the 2017 Forest Resources Survey Data of Yunnan Forestry Survey and Design Institute, which used the field survey method. The plots were laid out as squares, each with an area of 4 km^2^.

By regularly updating the survey plot data every 5 years, dynamic changes in forest resource growth and decline were obtained. We used the ecological quality formula of plots $Y=\sum_{i=1}^{7}W_{i}X_{i}$ to calculate a comprehensive score for each plot, where:

*i* is the evaluation index (1, 2, and 3 for types I, II, and III, respectively);
*X_i_* is the type score value of each evaluation index;
*W_i_* is the weight of each evaluation index.

We then assigned the type score value of each evaluation index according to the classification criteria (Table 3). The weights (as a percentage) were determined by via the expert scoring method for each evaluation index against the whole.

According to the comprehensive score of the plots, we used Equal Interval Natural Breaks Method to divide the range to four ecological quality grades (Table 4).

### 2.6. Habitat Quality Evaluation and Spatial Analysis

InVEST-Habitat Quality model [35] was used to evaluate snub monkey’s habitat quality based on LULC map from 2018 and biodiversity threat factors including villages, village roads, other non-forestry land, economic forest, cropland, and artificial construction [28,36]. We analyzed the correlation between villages, rural roads, and plots to get maximum distance thresholds of villages and rural roads in ArcGIS 10.6 and R [3,8]. Finally, we scored the parameters related to threat factor weights, suitability, and sensitivity of land use types and obtained threat factor attributes (Table 5) and sensitivity of land use types to threats (Table 6). We also applied Spatial Autocorrelation (Moran’s I) and Hot Spot Analysis (Getis-Ord Gi*) to explore the spatial variation characteristics of the influence of villages on Yunnan snub-nosed monkey habitat quality [37,38].

### 2.7. Impact of Village Development on Habitat Quality

We used the kernel density in ArcGIS to analyze the spatial pattern of the socio-economic development of the villages [39,40]. Then the impact of villages on the habitat quality of Yunnan snub-nosed monkeys was evaluated based on GIS spatial analysis.

## 3. Results

### 3.1. Analysis the Development of Villages

Our village development analysis revealed that more than half the villages (59.84%) were of Grade II (the second-lowest economic development grade). Grade III (just slightly higher economically developed than Grade II) represented 24.49% of the villages. The remaining Grades represented less than 7% of the villages each (Table 7).

**Figure 2:**
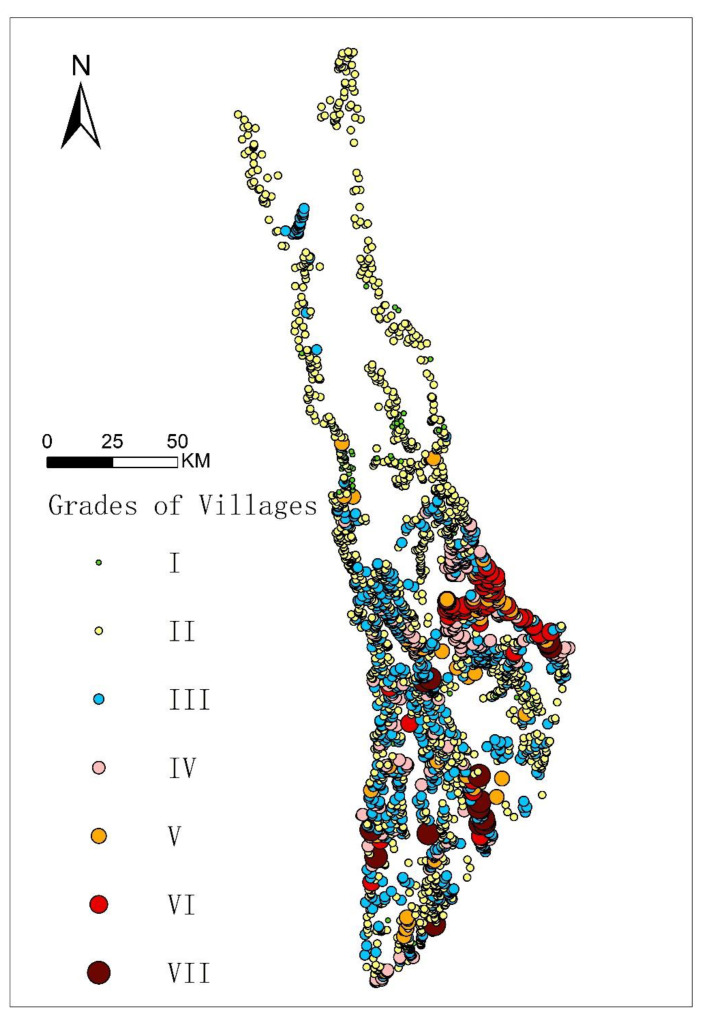
Distribution of villages and their grades in Northwest Yunnan.

**Figure 3:**
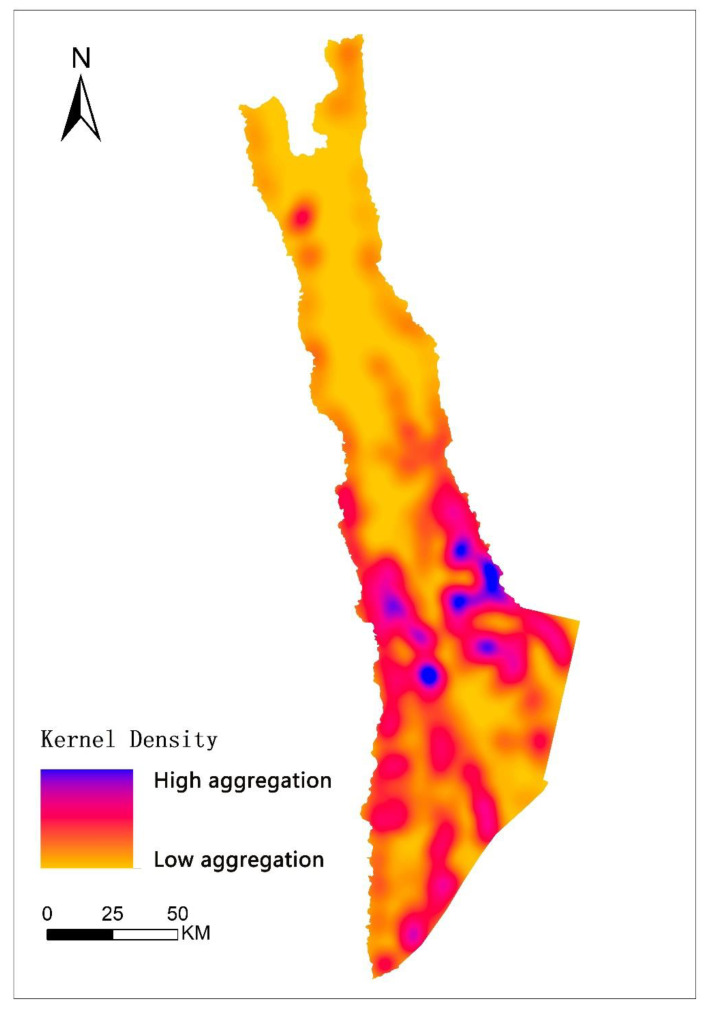
Distribution of kernel density in Northwest Yunnan.

Figure 2 shows higher density development of villages in the southern part of the study area than in the north, with the development trend showing a multi-center radiation pattern. This corresponds directly with a lower quality monkey habitat in the south than in the north as shown in Figure 3.

### 3.2. Analysis of the Impact of Village Development on Habitat Quality

**Figure 4:**
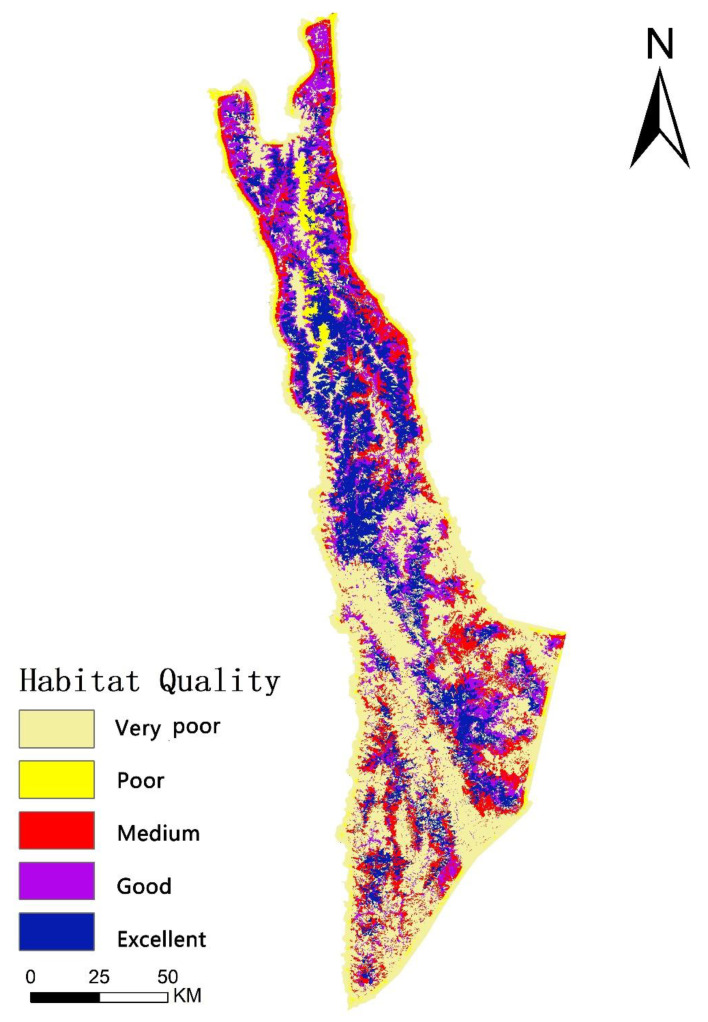
Spatial distribution pattern of habitat quality in 2018.

Figure 4 shows that habitat quality is higher in the northern part of the study area than in the southern part, that the development of villages in the north is less impactful on the habitat quality of snub-nosed monkeys than in the south, that ecological stability is poor, and that ecological protection needs to be prioritized.

The mean value of the habitat quality index for Yunnan snub-nosed monkeys in 2018 was 0.4679, representing a medium habitat quality. Areas of very poor-quality habitat represented over half the total area (7759.39 km^2^, or 51.94%). These very poor-quality habitats were mainly located in the outer and southern parts of the region and had villages densely distributed with socio-economic activities significantly degrading the habitat. The areas of good and excellent habitat quality were less than 30% of the total area and were mainly located in the northern part of the region. Those areas had more suitable habitat areas, less village distribution, and less human interference, resulting in slower habitat degradation (Table 8). Differing socio-economic development in the villages has caused differing degrees of habitat destruction, but overall socio-economic development has resulted in more low-quality habitat area and less high-quality habitat area.

### 3.3. Analysis of the Habitat Quality of the Yunnan Snub-Nosed Monkey

The analysis in Table 9 shows 12 monkey groups with a mean habitat quality value of 0.7408, which is relatively high for the overall habitat. The highest habitat quality was found in monkey group 11 (C11), with a value of 0.9047, while groups C3, C6, and C14 had the lowest habitat quality (Table 9).

## 4. Discussion

### 4.1. Impacts of Village Development on Habitat Quality

There were significant spatial differences in habitat quality distribution patterns. The spatial autocorrelation of habitat quality revealed characteristics of weak agglomeration distribution in the space. The statistical parameters of spatial analysis indicated that the global Moran’s I index of habitat quality was 0.0907, and the possibility of agglomeration distribution was less than 1%.

**Figure 5:**
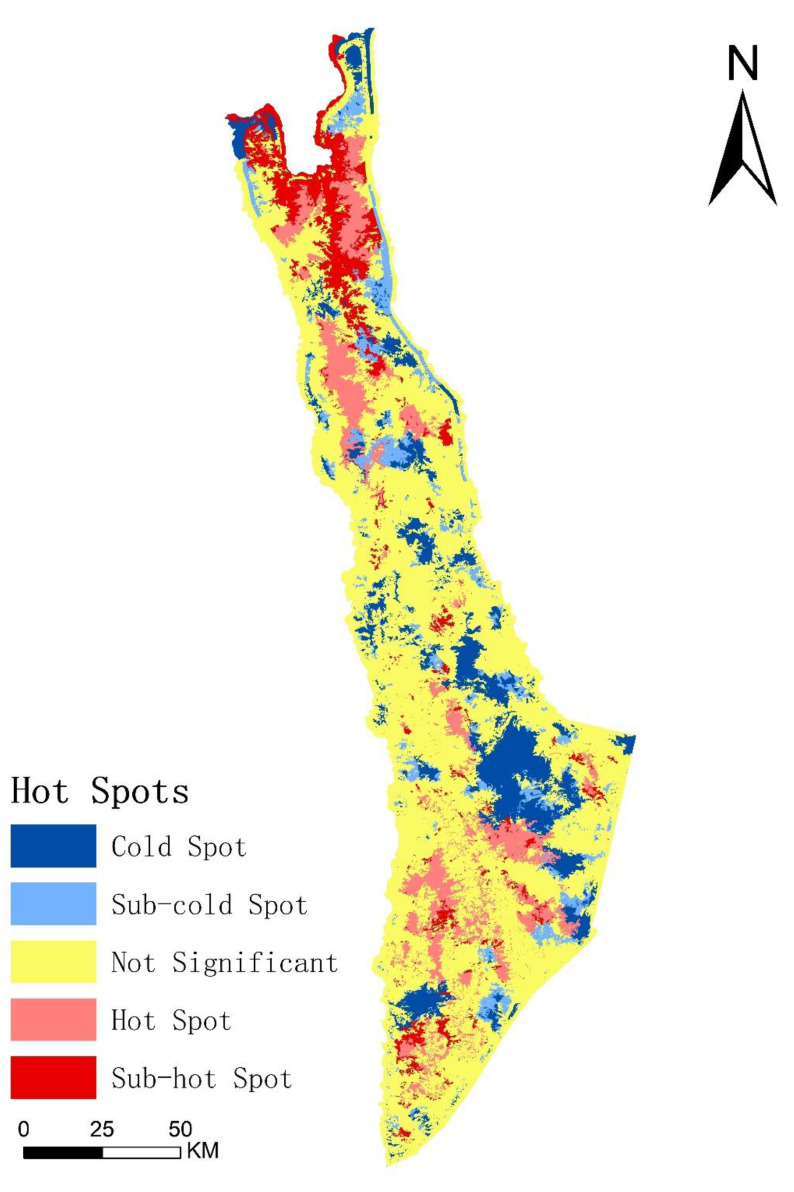
Hot spot analysis map of habitat quality in 2018.

Figure 5 reveals that the hot spots and sub-hot-spots of habitat quality were in the northern and southern region as opposed to the central region, and the habitat quality changed greatly, as formerly forested land was converted into non-forested land such as crop land and villages or converted into ecological restoration projects. Conversely, the cold spots and sub-cold-spots were mainly distributed in the central region, which is a relatively suitable habitat for Yunnan snub-nosed monkey with great conversation and little change in habitat quality. Comparing Figure 4 and Figure 5, we found that there was significant overlap between the Very Poor habitat quality area from Figure 4 and the Not Significant area from Figure 5.

If habitat or land use changes are representative of genetic, species, or ecosystem changes, then a low habitat quality will mean a decline in the biodiversity in the habitat and will mean habitat change unfavorable for species survival [16]. As the population of a village increases dramatically, people expand its boundaries, increase the use of cropland, and develop unused land to carry the increased population and provide food [7,41]. In addition, to develop the economy, people plant large amounts of economic forests, leading to habitat fragmentation [42]. Therefore, we need to carry out ecological restoration projects in these areas through cropland reforestation. We need to promote the restoration of the Huashan pine hemlock arrow bamboo forests, spruce fir forests, and mixed coniferous and broad-leaved forests to improve habitat quality [4]. Bamboo and lichen are important food sources for Yunnan snub-nosed monkeys [10]. Forest rangers should restore bamboo plants and lichen, which will provide adequate food conditions for the Yunnan snub-nosed monkeys.

### 4.2. Impacts of Village Development on Yunnan Snub-Nosed Monkeys

**Figure 6:**
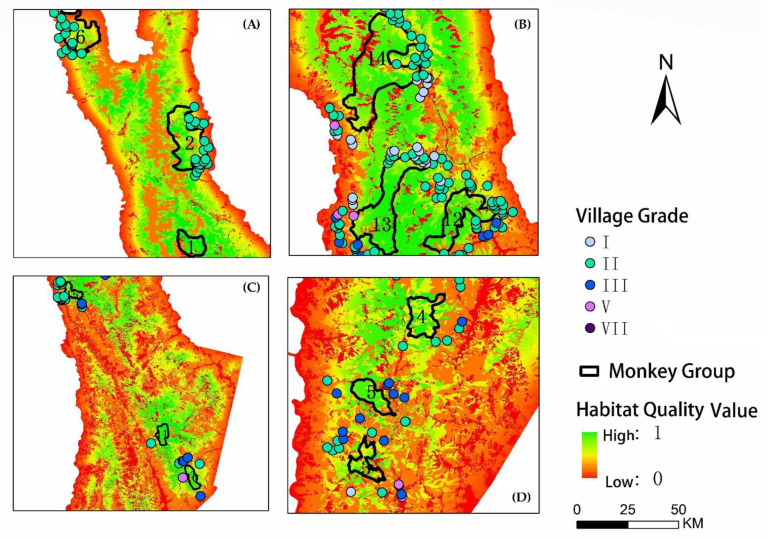
Zoom-in of four sections of the research area, showing the distribution of villages and habitat quality in relation to each monkey group. (**A**) Northern part of the area showing groups C1, C2, and C6. (**B**) North-central part of the area showing groups C12, C13, and C14. (**C**) South-central part of the area showing groups C10, C11, and C15. (**D**) Southern part of the area showing groups C3, C4, and C5.

Coordinating the relationship between the socio-economic development of the village and the protection of the ecological environment can promote a harmonious symbiosis between humans and Yunnan snub-nosed monkeys. In places where the socio-economic development of villages is lagging or in decline, local villagers currently rely on massive timber cutting and occupying forest land to develop agriculture and grazing to sustain their livelihoods, threatening the survival of monkeys. There were 10 villages (Zanzhuaro, Amu Shiguang, Muguang Aji, Sebu Feiha, Zaopo, Guilong, Cuding, Zengda, Yidoushe, and Lilinong) directly competing with the C3, C6, and C14 monkey groups (Figure 6). The government should implement poverty alleviation policies for these villages, removing the need of deforestation to drive economic development, instead developing monkey habitat–friendly eco-tourism and minority cultural industries.

Habitat quality, habitat patch size, and habitat connectivity mutually trade off against each other [43]. The habitat quality of the C3, C6, and C14 monkey groups is relatively low due to the development on villages. This indicates that village development had a strong influence on habitat quality, with the higher development level of a village translating to a lower habitat quality of the monkey group. We should enhance habitat connectivity and build up ecological corridors to promote gene exchange and conserve genetic diversity in these areas where connectivity with other monkey groups is impeded by agriculture and grazing land, roads, and villages. This is especially true for the isolated monkey groups (C3, C6, and C14) [4,5,6,44].

### 4.3. Implications for Conservation

In addition to protecting Yunnan snub-nosed monkeys through traditional measures such as ecological restoration, enhancing habitat connectivity, building corridors, relocation, and poverty alleviation, we need to take additional, creative measures. Illegal hunting is still an important factor threatening species conservation [45]. It is necessary to combat the illegal wildlife trade, curbing wildlife consumption through interventions and/or shifting consumption to more sustainable alternatives [46,47].

The government must provide ecological compensation to villages with high levels of ecological protection and restoration and establish ecological compensation mechanisms. These are a form of compensation for the damage cause to ecological functions and quality during development. The purpose of these forms of compensation is to improve the environmental quality of damaged areas or to be used to create new areas with similar ecological functions and environmental quality that can further protect, and sustainably use, ecosystem services [48,49]. We also need to strengthen science and education on biodiversity and wildlife conservation so that villagers can participate in species conservation and reap the benefits from it. In general, through a variety of measures, such as education, social influence, legal regulation, and behavioral facilitation, emphasis must be on multidisciplinary applications, multi-subject cooperation, and multi-scale considerations for species conservation [46,47].

The InVEST models is relatively mature and superior to transitional methods in spatial expression and dynamic research. We preliminarily discussed the improvement of adding quantitative village factors to the conventional model and have conducted analysis on the development on villages to include the habitat quality for Yunnan snub-nosed monkeys.

Due to the protection of some data and limited access to other data, the study area only has the distribution area of the Yunnan snub-nosed monkey in northwestern Yunnan. For example, data for some areas in Tibet province is lacking. The research is also lack dynamicity, integrity, and comprehensiveness. Data for some areas of Tibet will be supplemented in future work, and the influence of villages on the habitat quality of monkeys in the whole territory will be studied quantitatively. We also plan to improve the InVEST model parameters and strengthen parameter verification to obtain more effective variable factors, such as climate change factors. We also plan to further improve the variables that reflect the socio-economic development of villages, such as roads at all levels, population spatial distribution km grid data, nighttime lighting data, and GDP spatial distribution km grid data sets.

## 5. Conclusions

By using the smallest administrative scale in China—the village—we were able to accurately study the impact of village development on habitat quality, with focus on the Yunnan snub-nosed monkey. The modeling of habitat quality using the InVEST-Habitat Quality Model provided information about the relationship between the spatio-temporal distribution of habitat quality and potential biodiversity. The results of the habitat quality analysis indicate that decreases in habitat area and quality over time meant a decline in biodiversity persistence, resilience, and breadth and exacerbated the risk of declining species populations. In addition, the assessment of habitat quality based on species habitat suitability provides a new perspective to assess the impact of village developments on the habitat quality for the conservation of specific species. While we used the Yunnan snub-nosed monkey in our paper, these approaches can be applied for the conservation of other species.

## Figures and Tables

**Table 1:** Socio-economic data index of villages.

Category	Variable	Code	Category	Variable	Code
Natural resources (X1)	Area of commonly used cultivated land (km^2^)	X_11_	Economics (X3)	Total economic income (million yuan)	X_31_
	Paddy field area (km^2^)	X_12_		Income from farming (million yuan)	X_32_
	Dry land area (km^2^)	X_13_		Income from graziery (million yuan)	X_33_
	Area of cultivated land area per capita (km^2^)	X_14_		Forestry income (million yuan)	X_34_
	Area of economic fruit woodland (km^2^)	X_15_		Income of secondary and tertiary industries (million yuan)	X_35_
	Area of fruit woodland per capita (km^2^)	X_16_		Income per capita (yuan)	X_36_
Population (X2)	Rural population (people)	X_21_	Infrastructure (X4)	Distance to nearest station (km)	X_41_
	Agricultural population (people)	X_22_		Distance to nearest market (km)	X_42_
	Labor force (people)	X_23_		Cars (units)	X_43_
	Number of people in the primary industry (people)	X_24_		Agricultural transport vehicle (units)	X_44_
	Number of people with tertiary education and above (people)	X_25_		Tractors (units)	X_45_
	Number of secondary schools (people)	X_26_		Motorbike (units)	X_46_
	Number of primary school students (people)	X_27_	Energy resources (X5)	Biogas digester farmers (Households)	X_51_
	Number of people not attending school (people)	X_28_		Solar farmers (Households)	X_52_
			Education (X6)	Primary school enrollment rate (%)	X_61_
				Secondary school enrollment rate (%)	X_62_

**Table 2:** Classification standard of villages development grades.

Comprehensive Score Range/Z	(−∞,−0.5)	(−0.5,0)	(0,0.5)	(0.5,1)	(1.1.5)	(1.5,2)	(2,+∞)
Scale grades of villages	I	II	III	IV	V	VI	VII

**Table 3:** Plot evaluation indicators and weights.

Evaluation Index	Classification Criteria			Weight (%)
	I	II	III	
Forest naturalness	1, 2	3, 4	5	0.19
Forest community structure	1	2	3	0.18
Tree species structure	6, 7	3, 4, 5	1, 2	0.17
Total vegetation coverage	[70%, 100%]	[50%, 70%)	[0%, 50%)	0.14
Crown density	[0.7, 1.0]	[0.4, 0.7)	[0.2, 0.4)	0.13
Average tree height	[15.0, +∞)	[5.0, 15.0)	[0.9, 5.0)	0.13
Litter depth grade	1	2	3	0.06

**Table 4:** Classification criteria for the four ecological grades of plots.

Ecological Grade	Comprehensive Ecological Quality Score of Plots	Code
Excellent	<1.4	1
Good	1.4–1.8	2
Medium	1.8–2.2	3
Poor	>2.2	4

**Table 5:** The threat factors and related coefficients, including maximum effective distance of threats (km), weight, decay type.

Threat Factors	Maximum Effective Distance of Threats (km)	Weight	Decay Type
Village I	2	0.4	Exponential
Village II	2	0.5	Exponential
Village III	2	0.6	Exponential
Village IV	2	0.7	Exponential
Village V	2	0.8	Exponential
Village VI	2	0.9	Exponential
Village VII	2	0.95	Exponential
Village road	4	0.7	Linear
Other non-forestry land	1	0.6	Exponential
Economic forest	1	0.7	Exponential
Cropland	1	0.5	Exponential
Artificial construction	3	0.8	Exponential

**Table 6:** Sensitivity of land cover types to each threat factor. (Vil1 = Village I, Vil2 = Village II, Vil3 = Village III, Vil4 = Village IV, Vil5 = Village V, Vil6 = Village VI, Vil7 = Village VII, Vr = Village road, Onfl = Other non-forestry land, Ef = Economic forest, Cr = Cropland, Ac = Artificial construction.).

Land Cover Type Code	Land Cover Types	Habitat Suitability	Vil1	Vil 2	Vil 3	Vil 4	Vil 5	Vil 6	Vil 7	Vr	Onfl	Ef	Cr	Ac
1	other non-forest land	0	0	0	0	0	0	0	0	0	0	0	0	0
2	cold coniferous forest (Alpine coniferous forests)	0.6	0.7	0.75	0.8	0.9	0.9	0.9	0.9	0.95	0.4	0.3	0.4	0.8
3	shrublands	0.8	0.3	0.3	0.3	0.3	0.3	0.3	0.3	0.3	0.5	0.3	0.4	0.8
4	Armand pine and hemlock	1	0.9	0.9	0.9	0.9	0.9	0.9	0.9	0.9	0.5	0.4	0.5	0.9
5	barren land	0.2	0.5	0.5	0.5	0.5	0.5	0.5	0.5	0.5	0.2	0.1	0.1	0.2
6	broad-leaved forests	0.6	0.6	0.6	0.6	0.6	0.7	0.7	0.8	0.9	0.4	0.3	0.4	0.8
7	cropland	0	0	0	0	0	0	0	0	0	0	0	0	0
8	planted economic forests	0	0	0	0	0	0	0	0	0	0	0	0	0
9	water body	0.2	0.2	0.2	0.2	0.2	0.2	0.2	0.3	0.4	0.2	0.1	0.1	0.2
10	sclerophyllous evergreen broad-leaved forest	0.8	0.7	0.8	0.8	0.8	0.8	0.8	0.8	0.8	0.5	0.3	0.4	0.8
11	fir-spruce forest	1	0.8	0.8	0.8	0.8	0.8	0.8	0.8	0.8	0.5	0.4	0.5	0.9
12	warm coniferous forest (Yunnan pine forest)	0.2	0.3	0.3	0.3	0.3	0.4	0.4	0.4	0.4	0.2	0.3	0.1	0.2
13	coniferous broad-leaved mixed forest	1	0.8	0.8	0.8	0.8	0.8	0.8	0.85	0.85	0.5	0.4	0.5	0.9
14	Artificial construction	0	0	0	0	0	0	0	0	0	0	0	0	0

**Table 7:** The quantities and weight of grades of villages.

Village Grade	Quantities	Weight (%)
Grade I	138	4.20
Grade II	1874	59.84
Grade III	810	24.49
Grade IV	220	6.26
Grade V	74	2.45
Grade VI	57	2.18
Grade VII	21	0.58

**Table 8:** Percentage of area and mean value of habitat quality at grades in 2018.

Habitat Quality Grade	Value Interval	Area (km^2^)	Area Weight (%)
Very poor	[0, 0.2)	7759.39	51.94
Poor	[0.2, 0.4)	506.31	3.08
Medium	[0.4, 0.6)	2534.52	15.13
Good	[0.6, 0.8)	2585.69	13.24
Excellent	[0.8, 1)	3038.35	16.60

**Table 9:** The value of habitat quality in each monkey reserve.

Code of Monkey	Habitat Quality
C1	0.8107
C2	0.7113
C3	0.5943
C4	0.7139
C5	0.7723
C6	0.4982
C10	0.8074
C11	0.9047
C12	0.7653
C13	0.8238
C14	0.6943
C15	0.7931

## Data Availability

Data are available on request.
